# Responsiveness of the Spanish Version of Newcastle Stroke-Specific Quality of Life Measure (NEWSQOL)

**DOI:** 10.3390/ijerph181910034

**Published:** 2021-09-24

**Authors:** Concepción Soto-Vidal, Victoria Calvo-Fuente, Alfonso Muriel-García, Tomás Gallego-Izquierdo, Carlos González-Alted, Soraya Pacheco-da-Costa

**Affiliations:** 1 Department of Nursing and Physiotherapy, Universidad de Alcalá, 28871 Madrid, Spain; conchi.soto@uah.es (C.S.-V.); alfonso.muriel@uah.es (A.M.-G.); tomas.gallego@uah.es (T.G.-I.); soraya.pacheco@uah.es (S.P.-d.-C.); 2 Medical Director State Reference Center for Brain Damage Care, 28034 Madrid, Spain; cgonzaalted@imserso.es

**Keywords:** quality of life, stroke, responsiveness, physical therapy, NEWSQOL

## Abstract

Objective: To evaluate the responsiveness of the Spanish version of the Newcastle Stroke-specific Quality of Life measure (NEWSQOL) to assess quality of life in Spanish people after suffering a stroke. Design: A prospective observational study was conducted to assess the responsiveness of the Spanish version of NEWSQOL. The sample contained 128 patients who filled in the questionnaires before and after a physical therapy intervention. The responsiveness was assessed with *p*-values using the effect size (ES) and the standardized response means (SRMs) of the change. Besides, two other external criteria were used to distinguish patients who improved with the treatment from those who remained stable. This classification was based on one functional independence measure (the Barthel Index) and one disability measure (the modified Rankin Scale). Results: There was a statistically significant correlation (Spearman’s coefficient = *p* < 0.01) between the domains of the Spanish version of NEWSQOL in relation to the Barthel Index and the modified Rankin Scale. All domains showed between marked-to-mild change responsiveness except sleep and relationships; mobility (ES 0.66 and SRM 0.92) and activities of daily living (ES 0.75 and SRM 0.87) were markedly responsive; communication (ES 0.38 and SRM 0.61) was moderately responsive; and pain, vision, cognition, feelings, emotions and fatigue were mildly responsive (ES 0.21–0.41 and SRM 0.23–0.44). Conclusion: The Spanish version of NEWSQOL shows between marked and mild responsiveness to measure the perception of QoL in post-stroke patients. Therefore, its use can be suitable for evaluation studies, clinical trials and clinical practice.

## 1. Introduction

The World Health Organization defines stroke as “the rapid onset of clinical symptoms of focal or global brain dysfunction, which lasts longer than 24 h or leads to death, with no other apparent cause than a vascular injury” [1]. The number of stroke patients around the world has increased considerably in recent decades [2], especially since the global pandemic started, where stroke arose as a complication of COVID-19, although up to now, its incidence remains unknown [3,4]. Clinical manifestations are wide and varied, including motor, sensory, perception and cognitive disturbances; urinary and faecal incontinence; swallowing and visual problems; and pain, communication and behavioural alterations [5,6,7,8,9,10,11,12,13,14]. Although motor disturbances might be responsible for a high rate of disability, sometimes patients report other important aspects [15], such as difficulties in performing activities of daily living (ADL), risk of falls and a negative impact on their quality of life (QoL) [16,17,18,19,20,21].

Health-related quality of life (HRQoL) measures have been of most importance, and it is essential to assess patients’ perceptions. A valid way to measure such subjective sensations is through psychometrically validated questionnaires that, objectively, collect information on other aspects of the disease, its impact on the patient’s QoL and changes after a therapeutic approach [22,23].

The Newcastle Stroke-specific Quality of Life measure (NEWSQOL) is a specific questionnaire to measure the QoL of patients who suffered a stroke. It was developed and validated by Buck et al. [24]. It is different from other questionnaires because it includes domains of vision, cognition and communication, and it can used for patients with ischaemic or haemorrhagic stroke and motor aphasia. In the Spanish version of NEWSQOL [25], the psychometric properties of feasibility, validity, reliability and ceiling and floor effects are tested. However, NEWSQOL responsiveness is not published at the moment, neither in the original version nor in the Spanish version.

Responsiveness is defined as the “ability to detect changes that are significant or clinically important” [26]. It is an essential psychometric property in any questionnaire in order to detect positive or negative changes that might occur after a therapeutic intervention or other changes regarding a disease, and should be considered a key feature of assessment instruments designed to measure longitudinal change over time [27,28]. Assessing the responsiveness of an instrument is of special importance, both in clinical practice and in research studies, to evaluate the effectiveness of health interventions.

Therefore, the aim of this study was to report the responsiveness of the Spanish version of NEWSQOL to assess the quality of life in Spanish post-stroke people who undergo physical therapy.

## 2. Materials and Methods

A multicenter longitudinal observational study was conducted. Patients who had survived a stroke were recruited by physical therapists from 3 different centres in Spain between September 2012 and December 2018: Ramon and Cajal Hospital, the Brain Damage State Centre (CEADAC) and the Institute of Neurological Diseases (IEN). The inclusion criteria were patients who had suffered a stroke and were older than 18 years and the time after stroke of 1 month to 2 years. The exclusion criteria were subjects who had serious or potentially terminal comorbidities, presented other neurological or neuromuscular diseases that had repercussions on their QoL, were diagnosed with serious psychiatric diseases, were dependent for ADL before the stroke or had moderate or severe cognitive deterioration according to Pfeiffer’s questionnaire [29].

All participants signed a written informed consent form before their inclusion in the study, and the data collected from each participant were associated with a code for anonymity guarantee.

The study was approved by the Ramon and Cajal University Hospital Research Ethics Committee in Madrid, Spain (protocol no. 120/11).

The demographic data and clinical history of patients were collected at baseline. The Spanish version of the NEWSQOL questionnaire (Supplementary S1), the Barthel Index (BI) and the modified Rankin Scale (mRS) were fulfilled twice by neurological physical therapists: at baseline and at 6 months after a physical therapy intervention, which consisted of motor control techniques, 3 sessions per week, 40 min each session.

NEWSQOL includes 56 items divided into 11 domains: mobility (items 1–9), ADL/self-care (items 10–17), pain/sensation (items 18–20), vision (items 21–22), cognition (items 23–27), communication (items 28–31), feelings (items 32–37), interpersonal relationships (items 38–43), emotions (items 44–47), sleep (items 48–53) and fatigue (items 54–56). Each item can be answered on four levels of involvement: none, a little, moderately or a lot. The minimum score for each item is 0 points (low effect), and the maximum is 3 points (maximum effect), and they are not significant individually. The results of each domain are obtained by the sum of the scores of the items; higher values indicate greater impact on the quality-of-life perception. The author of the original questionnaire does not recommend adding the scores obtained in the domains to achieve a global score [24] (Table 1).

The mRS is used for measuring the degree of disability or dependence in ADL of people who have suffered a stroke. The scale is reported as ranging from 0 (no symptoms) to 6 (death), and it is valid, reliable and easy to use [30].

The BI is a generic instrument developed to assess functional independence in ADL, generally in post-stroke patients [31]. It is valid and reliable, and easy to use, and it shows better responsiveness than other generic scales for stroke patients [32]. It ranges from 0 to 100 points, and the better the score, the more independent the person [33].

To assess the responsiveness of a questionnaire, it is necessary to use some criteria to prove whether patients have experienced change throughout the study, and it is recommended to use independent criteria [34]. In this study, the change scores of the mRS and BI were used as two external anchor criteria. The first criterion was based on the change between categories in the mRS at the beginning and at the end of the 6 months of follow-up [35]. Improvement = improvement of at least one level; stable = unchanged; and impairment = loss of at least one level were considered.

Regarding BI anchor criteria, we used the minimally clinically important difference (MCID) of 1.85 points estimated by Hsiech et al. [36]. The results were a reference value of 9.25 on a 100 point-scale. They were interpreted as follows: improvement = increase of 9.25 points or more; deterioration = decrease of 9.25 points or more; and stable = change (increase/decrease) less than 9.25 points. No distinction was made between “some” and “big” changes. The average change was calculated as average change score seen in the cohort defined to be responders; minimally detectable change = equal to the upper value of the 95% confidence interval for average change scores seen in the cohort defined to be nonresponders; and change difference = the difference of the average change score for responders and nonresponders.

Correlations between the score changes of the different measures were evaluated with Spearman’s rank correlation coefficient. In addition, questionnaire responsiveness was evaluated using two statistical methods: the effect size (ES) and the change standardized response mean (SRM) in the scores at the assessments for each domain of the questionnaire. Baseline scores were compared with scores obtained 6 months later, using the Wilcoxon test because no normality score in the Shapiro test was found. The ES refers to the mean change in the score divided by the score domain standard deviation (SD) at baseline. The denominator standardizes the difference by transforming the absolute difference into baseline standard deviation units. The SRM is equivalent to the change in score over a period of time divided by the change in SD. Both the ES and the SRM were interpreted as follows: <0.2 as non-responsive, 0.2–0.5 as mildly responsive, 0.51–0.7 as moderately responsive and ≥0.7 as markedly responsive to change [37].

The data was statistically analyzed using SPSS version 26 for Windows. The results were considered statistically significant for *p*-value < 0.05 (two-tail test).

## 3. Results

**Figure 1:**
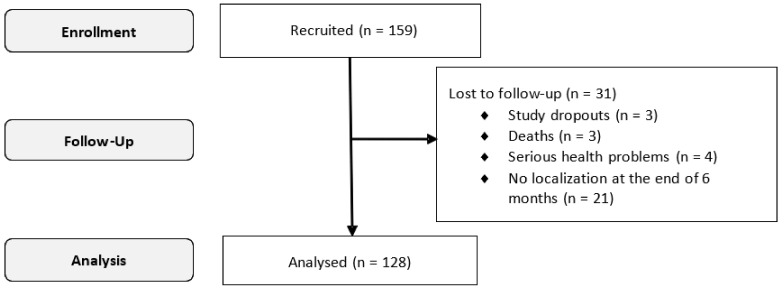
Flowchart: progress of patients through the study.

A total of 159 subjects were recruited for the study, of which 128 subjects filled in the Spanish version of NEWSQOL in both assessments and were included for responsiveness validity of the questionnaire (Figure 1).

Table 2 shows the socio-demographic and clinical characteristics of the sample. Most of the subjects were middle-aged (49 years old) men (51.6%) who were under sick leave (68.0%) and had help from their families (92.9%). Comorbidities in the study sample, such as hypertension (31.4%), diabetes (13.2%) and others (23.2%), were common. The mean baseline score for the BI was 64.7 (31.2) and for the mRS was 4 (2.5; 4).

During the 6-month follow-up period, the results of each domain of the Spanish version of NEWSQOL showed improvement in terms of the mean score (SD), as well as the overall scores of the BI and the mRS (Table 3).

Correlation was demonstrated by the score changes of each domain of the Spanish version of NEWSQOL and the measures considered anchor criteria (Table 4). Spearman’s correlation coefficient showed a statistically significant correlation for the results of each of the domains of the Spanish version of the NEWSQOL questionnaire with the BI, except the domains of sleep and fatigue. A strong correlation was shown with the dimensions of mobility (r = −0.883; *p* < 0.01) and limitation for ADL (r = −0.808; *p* < 0.01) and moderate correlation with the dimensions of feelings (r = −0.382; *p* < 0.01), interpersonal relationships (r = −0.251; *p* < 0.01), communication (r = −0.277; *p* < 0.01) and vision (r = −0.197; *p* < 0.01). The correlation was low for pain (r = −0.146; *p* < 0.05), cognition (r = −0.184; *p* < 0.05) and emotion (r = −0.322; *p* < 0.05).

In relation to the mRS, there was a high correlation with the dimensions of mobility (r = 0.871; *p* < 0.01), ADL (r = 0.770; *p* < 0.01) and feelings (r = 0.469; *p* < 0.01). The correlation was moderate with dimensions of pain (r = 0.244; *p* < 0.01), cognition (r = 0.207; *p* < 0.01), interpersonal relationships (r = 0.305; *p* < 0.01), communication (r = 0.240; *p* < 0.01), vision (r = 0.186; *p* < 0.01) and fatigue (r = 0.218; *p* < 0.01). It showed no correlation with the domains of sleep and emotion.

Regarding mRS scores, 75% of the patients improved (96 responders) compared to the data of the BI as anchor criteria, where 49% patients improved (63 responders), and any of them suffered deterioration. Nevertheless, there was a percentage of 25% in the case of the mRS and 51% in the case of the BI where the patients’ improvement did not reach the pre-established levels (nonresponders). Table 5 shows the results of each domain of the Spanish version of NEWSQOL for each subgroup and for each of the external criteria, mRS and BI. In the analysis based on anchor criteria, the values of both the ES and the SRM were higher either for the average change or for the change difference. For the Spanish version of NEWSQOL, the average change, in relation to the BI, showed a change in every domain and a correlation was established between the magnitude of average change and the statistics associated with responsiveness. The same pattern can could observed when the mRS data were used as the anchor criteria.

The correlation of both showed that the results had been modified, in all domains, between initial and final moments. Observing the average change and change difference, the results were higher for the average change, as expected, since it focuses specifically on responder patients. In the change difference were included responder and nonresponder patients, and it was observed that two domains, sleep and interpersonal relationships, were not modified, resulting in these domains being interpreted as non-responsive with the statistical methods of the ES and the SRM. The highest values in the average change and change difference corresponded with each other and with the results of the ES and the SRM that indicated greater responsiveness to change.

## 4. Discussion

As far as the authors know, this is the first study for assessing the responsiveness of NEWSQOL since its development in 2004 [24]. This is not an isolated phenomenon; there are other quality-of-life (QoL) questionnaires widely used in post-stroke patients, both in clinical practice and in research studies that are culturally adapted to other languages other than the original where responsiveness is not studied either [38].

There is consensus among researchers that the effectiveness of a therapeutic intervention is important to measure it not only by survival time but also by the QoL perceived by the subject during that time. However, the lack of specific instruments, with psychometric properties studied rigorously, has been a constant inconvenience when working with post-stroke patients [39]. Therefore, clinicians try to choose the best option among the different instruments to evaluate patients´ perception about their health status changes. In addition, the selection and responsiveness of an instrument that best suits the characteristics of the disease or the pathological process is also important. Responsiveness is suggested as a basic criterion for the choice of instrument to be used when assessing a therapeutic intervention [40], although there is currently no consensus in the literature on the concept of responsiveness to change [27]. There are different statistical methods to evaluate this psychometric property, but no gold standard has been determined for a significant change in the QoL of post-stoke patients. It is most advisable to use multiple change criteria based on clinical anchors [26].

The sample size of this study was bigger than the ones in most measurement instrument validation studies on post-stroke patients. The clinical and socio-demographic characteristics were similar to other validation studies [41,42,43]. All participants had suffered a stroke between 3 and 9 months before being included in the study, and this period is within the range frequently showed in other validation studies of instruments for measuring the QoL after suffering a stroke [44].

Most responsiveness studies of specific instruments for patients who have suffered a stroke use a single statistical method, usually the ES or the SRM, and do not do so in relation to external measures [42,44,45,46,47], so it is not possible to assess in parallel whether these patients have undergone changes and the extent of those changes [26]. In this study, two anchor criteria were used; on the one hand, the patients who had improved were identified, and on the other hand, those who had remained stable were identified. This classification was based on a measure of disability (mRS) and a measure of functional independence (BI). The comparison with an external measuring instrument may allow researchers to obtain more reliable results [26,43]. The authors chose the mRS and the BI as two of the most commonly used standard instruments for post-stroke patients [48] also used in responsiveness studies on other QoL instruments [42,44,47,49].

The results found in this study present similarities, but also differences with those found in other responsiveness studies. The Spanish version of the NEWSQOL questionnaire was able to detect the recovery of patients during a physical therapy intervention’s 6-month follow-up period in most domains, except for sleep and interpersonal relationships. It showed marked responsiveness for the domains of mobility and ADL, moderate responsiveness for communication and mild responsiveness for the domains of pain, vision, cognition, feelings, emotion and fatigue. Comparing responsiveness to other QoL-specific measurement instruments for stroke, the Escala de calidad de vida para el Ictus (ECVI-38) [39] showed between mild and marked responsiveness, although it does not include the domains of pain, vision, sleep and fatigue, unlike NEWSQOL. The Danish version of the Stroke-Specific Quality of Life scale (SS-QOL) [47] showed mild-to-moderate responsiveness for most domains, except for the domains of personality, energy, upper extremity function and thinking, which were non-responsive. This scale does not include domains of pain, feelings, emotions and sleep. In the French version of the SS-QOL, 9 of the 12 domains showed mild-to-moderate responsiveness and only the energy domain was markedly responsive according to the SRM but not the ES [41]. HRQOLISP-40 showed marked global responsiveness within the physical sphere but not in the other domains [42]. The Stroke and Aphasia Quality of Life Scale-39 (SA-QOL), Singaporean version, showed mild responsiveness globally, and the changes were only significant for patients without aphasia [44].

Regarding generic HRQoL measures, two of the most commonly used in stroke trials are SF-36 and SF-12. SF-12 showed mild responsiveness for the physical component and moderate responsiveness for the mental component in a study that examined the responsiveness among stroke patients [50]. In some studies carried out with post-stroke patients, EuroQol 5 dimensions 5 Levels (EQ-5D-5L) was used to measure the QoL, and it showed moderate responsiveness in the global score. The study was limited to responders, avoiding nonresponders [26,51]. Generic QoL measures are common features in many stroke studies despite the disadvantage that most of such measures are less likely to assess aspects of life that have importance for post-stroke patients, such as fatigue, sleep, cognition, communication, feelings, emotions, vision and interpersonal relationships. These findings support some statements that generic QoL measures are less responsive to change compared to stroke-specific ones [52].

The fact that a measuring instrument is responsive is of vital importance for studies in which there is a special interest in patients´ evolution over time and comparing therapeutic interventions about the patients´ perception about their health status—hence the importance of not using instruments that merely assess isolated aspects of recovery or do not allow for the inclusion of the entire population. NEWSQOL is different from others questionnaires because it includes domains of vision, cognition and communication and it can used for patients with ischaemic or haemorrhagic stroke and motor aphasia.

As a limitation, we did not make any distinction between “some” and “big” changes” of the scores.

## 5. Conclusions

In conclusion, the Spanish version of NEWSQOL showed between marked and mild responsiveness to measure the perception of the QoL in post-stroke patients who received a physical therapy intervention for 6 months. Therefore, its use can be suitable for evaluation studies, clinical trials and clinical practice.

## Figures and Tables

**Table 1:** Scoring NEWSQOL.

Domains	Mobility	ADL/Self-Care	Pain/Sensation	Vision	Cognition	Communication	Feelings	IR	Emotion	Sleep	Fatigue	NEWSQOL
Items	1–9	10–17	18–20	21–22	23–27	28–31	32–37	38–43	44–47	48–53	54–56	
	1	1	1	1	1	1	1	1	1	1	1	
	2	2	2	2	2	2	2	2	2	2	2	
	3	3	3		3	3	3	3	3	3	3	
	4	4			4	4	4	4	4	4		
	5	5			5		5	5		5		
	6	6					6	6		6		
	7	7										
	8	8										
	9											
Range	0–27	0–24	0–9	0–6	0–15	0–12	0–18	0–18	0–12	0–18	0–9	No score

ADL: activities of daily living; IR: interpersonal relationships. None: 0; a little: 1; moderately: 2; a lot: 3.

**Table 2:** Socio-demographics and clinical characteristics of the sample.

Sex (%)	
Women	62 (48.4%)
Men	66 (51.6%)
Age (median (p25; p75))	49 (43; 61.5)
Type of stroke (%)	
Ischaemic	69 (53.9%)
Haemorrhagic	59 (46.1%)
Affected hemibody (%)	
Left	57 (44.3%)
Right	59 (46.1%)
Both	12 (9.4%)
Time after stroke (months) (median (p25; p75))	6 (3; 9)
Employment situation (%)	
Active	1 (0.8%)
Unemployed	10 (7.8%)
Retired	30 (23.4%)
Sick leave	87 (68.0%)
Comorbidities (%)	
Arterial hypertension	50 (39.1%)
Diabetes	21 (16.4%)
Other	37 (28.9%)
Family situation (%)	
Family help	119 (92.9%)
Lives alone	1 (0.8%)
Institutionalized	8 (6.3%)
BI	64.7 (31.20)
mRS (median (p25; p75))	4 (2.5; 4)

BI: Barthel Index; mRS: modified Rankin Scale.

**Table 3:** Mean change in scores of the Spanish version of the NEWSQOL questionnaire, BI and mRS.

NEWSQOL (n = 128)	Pretreatment Mean Score	SD	Posttreatment Mean Score	SD	*p* (Wilcoxon Test)
Mobility	15.7	8.8	9.8	8.4	0.000
ADL/self-care	15.8	6.9	10.6	7.9	0.000
Pain/sensation	2.1	2.4	1.4	1.8	0.000
Vision	2.1	1.9	1.7	1.7	0.006
Cognition	6.0	4.6	4.6	4.3	0.000
Communication	5.8	4.1	4.3	3.8	0.000
Feelings	14.7	4.3	13.0	4.8	0.000
Interpersonal relationships	4.0	4.1	3.6	3.9	0.120
Emotion	6.0	3.1	4.7	2.8	0.000
Sleep	6.4	4.9	5.8	5.1	0.073
Fatigue	2.9	2.6	2.3	2.5	0.002
**Barthel Index**	64.7	31.2	77.8	27.1	0.000
	**Pretreatment Mean Score**	**IQR**	**Posttreatment Mean Score**	**IQR**	
**Modified Rankin Scale** Median (p25; p75)	4	2.5; 4	2	1; 4	0.000

ADL: activities of daily living; SD: standard deviation; IQR: interquartile range.

**Table 4:** Correlation between change scores of studied measures (Spearman’s correlation coefficient).

Domain of NEWSQOL	BI	mRS
Mobility	−0.883 **	0.871 **
ADL/self-care	−0.808 **	0.770 **
Pain/sensation	−0.146 *	0.244 **
Vision	−0.197 **	0.186 **
Cognition	−0.184 *	0.207 **
Communication	−0.277 **	0.240 **
Feelings	−0.382 **	0.469 **
Interpersonal relationships	−0.251 **	0.305 **
Emotion	−0.322 *	0.255
Sleep	−0.170	−0.041
Fatigue	−0.151	0.218 **

mRS: modified Rankin Scale; BI: Barthel Index; ADL: activities of daily living. ** *p* ≤ 0.01; * *p* ≤ 0.05.

**Table 5:** Responsiveness statistics for the Spanish version of NEWSQOL and clinical measures by external criteria.

NEWSQOL Domain	BI-Based External Criterion			mRS-Based External Criterion			Effect Size (ES)	Standardized Response Mean (SRM)
	Average Change	Minimum Change Detectable	Change Difference	Average Change	Minimum Change Detectable	Change Difference		
	**n = 63**	**n= 65**	**n = 128**	**n = 96**	**n = 32**	**n = 128**	**n = 128**	**n = 128**
Mobility	−8.7	−4.1	−5.5	−6.8	−4.1	−4.2	0.66	0.92
ADL/self-care	−7.3	−4.4	−4.1	−6.5	−3.1	−5.1	0.75	0.87
Pain/sensation	−0.9	1.2	−0.3	−0.6	2.2	0.8	0.33	0.37
Vision	−0.3	1.0	−0.3	−0.4	0.9	0.0	0.21	0.23
Cognition	−1.6	2.1	−0.5	−1,5	2.5	−0.5	0.29	0.37
Communication	−1.9	1.9	−0.6	−1.7	2.1	−0.5	0.38	0.61
Feelings	−2.1	2.6	−0.6	−1.9	3.2	−0.4	0.41	0.41
Interpersonal relationships	−0.5	1.4	0.1	−0.2	2.2	0.9	0.11	0.13
Emotion	−1.3	2.0	0.0	−1.2	2.4	0.1	0.41	0.44
Sleep	−0.1	2.3	0.9	−0.4	2.8	0.7	0.13	0.11
Fatigue	−1.1	−0.8	−0.9	−0.7	1.3	−0.3	0.25	0.25

ADL: activities of daily living; BI: Barthel Index; mRS: modified Rankin Scale.

## Data Availability

The data presented in this study are available on request from the corresponding author. The data are not publicly available due to the ethical declaration issue.

## Supplementary Materials

The following are available online at https://www.mdpi.com/article/10.3390/ijerph181910034/s1. Spanish version of NEWSQOL.
