# Supplementary material for: Responsiveness of the Spanish Version of Newcastle Stroke-Specific Quality of Life Measure (NEWSQOL)
Source: Int J Environ Res Public Health. 2021 Sep 24;18(19):10034. doi: 10.3390/ijerph181910034 (PMC8507955; doi:10.3390/ijerph181910034)
Supplement: Supplementary file 1 [file ijerph-18-10034-s001.zip › ijerph-1322985-supplementary.pdf]

Spanish version of NEWSQOL.

**Movilidad (0-27)**	
1. ¿Se desplaza en silla de ruedas?	No: 0 / Puntualmente: 1 / Algunas veces: 2 / Siempre: 3
2. ¿Tiene dificultad para caminar 800 metros?	No: 0 / Algo: 1 / Mucha: 2 / No puedo: 3
3. ¿Tiene dificultad para subir o bajar pendientes?	No: 0 / Algo: 1 / Mucha: 2 / No puedo: 3
4. ¿Camina con bastón, andador o sujetándose a algo?	No: 0 / Algunas veces: 1 / Siempre: 2 / No puedo caminar: 3
5. ¿Piensa que camina despacio?	No: 0 / Bastante: 1 / Mucho: 2 / No puedo caminar: 3
6. ¿Tiene dificultad para subir o bajar escaleras solo?	No: 0 / Algo: 1 / Mucha: 2 / No puedo: 3
7. ¿Tiene dificultad para agacharse?	No: 0 / Alguna: 1 / Mucha: 2 / No puedo: 3
8. ¿Se siente inestable cuando está de pie?	No: 0 / Bastante: 1 / Mucho: 2 / No puedo mantenerme: 3
9. ¿Tiene dificultad para mantenerse de pie durante un tiempo?	No: 0 / Poca: 1 / Mucha: 2 / No puedo: 3
**Actividades de la vida diaria (0-24)**	
10. ¿Tiene dificultad con las tareas domésticas?	No: 0 / Alguna: 1 / Mucha: 2 / No puedo: 3
11. ¿Tiene dificultad para cocinar?	No: 0 / Alguna: 1 / Mucha: 2 / No puedo: 3
12. ¿Le resulta difícil preparar la comida, cortar una rebanada de pan o cortar verduras?	No: 0 / Algo: 1 / Mucho: 2 / No puedo: 3
13. ¿Tiene dificultad para hacerse cargo de las compras?	No: 0 / Algo: 1 / Mucha: 2 / No puedo: 3
14. ¿Le resulta difícil usar el transporte público?	No: 0 / Algo: 1 / Mucho: 2 / No puedo: 3
15. ¿Le resulta difícil asearse solo?	No: 0 / Algo: 1 / Mucho: 2 / No puedo: 3
16. ¿Le resulta difícil vestirse, incluidos cremalleras y botones?	No: 0 / Algo: 1 / Mucho: 2 / No puedo: 3
17. ¿Tiene dificultad para entrar o salir de la bañera/ducha solo?	No: 0 / Alguna: 1 / Mucha: 2 / No puedo: 3
**Dolor (0-9)**	
18. ¿Tiene dolor?	No: 0 / Un poco: 1 / Bastante: 2 / Mucho: 3
19. ¿Con qué frecuencia sufre dolor?	Nunca: 0 / Puntualmente: 1 / Algunas veces: 2 / Siempre: 3
20. ¿Tiene dificultad para coger cosas pequeñas?	No: 0 / Alguna: 1 / Mucha: 2 / No puedo: 3
**Visión (0-6)**	
21. ¿Tiene problemas de visión?	No: 0 / Leve: 1 / Moderado: 2 / Grave: 3
22. ¿Tiene dificultad para leer?	No: 0 / Algo: 1 / Mucha: 2 / No puedo: 3

Cognición (0-15)

23. ¿Tiene dificultad para resolver problemas o tomar decisiones?	No: 0 / Poca: 1 / Bastante: 2 / Mucha: 3
24. ¿Hay veces que olvida lo que ha dicho o lo que le dicen?	No: 0 / Puntualmente: 1 / Algunas veces: 2 / Siempre: 3
25. ¿Le resulta difícil concentrarse?	No: 0 / Un poco: 1 / Bastante: 2 / Mucho: 3
26. ¿Se le siguen olvidando cosas?	No: 0 / Puntualmente: 1 / Algunas veces: 2 / Siempre: 3
27. ¿Le resulta difícil pensar con claridad?	No: 0 / Un poco: 1 / Bastante: 2 / Mucho: 3

Comunicación (0-12)

28. ¿Siente como si su pronunciación no fuese correcta?	No: 0 / Un poco: 1 / Bastante: 2 / Mucho: 3
29. ¿Tiene dificultad para hacerse entender?	No: 0 / Un poco: 1 / Bastante: 2 / Mucho: 3
30. ¿Hay ocasiones en las que tiene dificultad para expresarse?	No: 0 / Puntualmente: 1 / Algunas veces: 2 / Siempre: 3
31. ¿Tiene dificultad para escribir?	No: 0 / Alguna: 1 / Mucha: 2 / No puedo: 3

Sentimientos (0-18)

32. ¿Se siente menos independiente de lo que era?	No: 0 / Un poco: 1 / Bastante: 2 / Mucho: 3
33. ¿Ha variado el ictus la percepción de sí mismo?	No: 0 / Un poco: 1 / Bastante: 2 / Mucho: 3
34. ¿Hasta qué punto diría que su vida ha cambiado?	No: 0 / Un poco: 1 / Bastante: 2 / Mucho: 3
35. ¿Se siente deprimido?	No: 0 / Puntualmente: 1 / A veces: 2 / Siempre: 3
36. ¿Se siente inútil?	No: 0 / Un poco: 1 / Bastante: 2 / Completamente: 3
37. ¿Siente que tiene menos control sobre lo que está pasando en su vida?	No: 0 / Un poco: 1 / Mucho menos: 2 / Ningún control: 3

Relaciones interpersonales (0-18)

38. ¿Discute más con sus amigos íntimos o familiares?	No: 0 / Un poco: 1 / Mucho: 2 / A todas horas: 3
39. ¿Hay más tensión en la relación con su pareja?	No: 0 / Un poco: 1 / Bastante: 2 / Muchísima: 3
40. ¿Interfiere el ictus en su vida sexual? ¿Cuánto?	No: 0 / Un poco: 1 / Bastante: 2 / Mucho: 3
41. ¿Está más irritable?	No: 0 / Un poco: 1 / Bastante: 2 / Mucho: 3
42. ¿Es menos tolerante?	No: 0 / Un poco: 1 / Bastante: 2 / Mucho: 3
43. ¿Le pone nervioso quedar con gente?	No: 0 / Un poco: 1 / Bastante: 2 / Mucho: 3

Emociones (0-12)

44. ¿Se nota más sensible?	No: 0 / Un poco: 1 / Bastante: 2 / Mucho: 3
45. ¿A veces llora por la mínima cosa?	No: 0 / Puntualmente: 1 / A veces: 2 / Siempre: 3

46. ¿Está preocupado porque podría tener otro ictus?	No: 0 / Un poco: 1 / Bastante: 2 / Mucho: 3
47. ¿Le preocupa volverse dependiente de otras personas?	No: 0 / Un poco: 1 / Bastante: 2 / Muchísimo: 3

Sueño (0-18)

48. ¿Tiene problemas para dormir por la noche?	No: 0 / Puntualmente: 1 / A veces: 2 / Siempre: 3
49. ¿Tiene dificultades para conciliar el sueño?	No: 0 / Puntualmente: 1 / A veces: 2 / Siempre: 3
50. ¿A veces se despierta demasiado temprano?	No: 0 / Puntualmente: 1 / A veces: 2 / Siempre: 3
51. ¿Encuentra que necesita descansar mucho?	No: 0 / Puntualmente: 1 / A veces: 2 / Siempre: 3
52. ¿Se siente agotado?	No: 0 / Puntualmente: 1 / A veces: 2 / Siempre: 3
53. ¿Siente que le falta energía?	No: 0 / Puntualmente: 1 / A veces: 2 / Siempre: 3

Fatiga (0-9)

54. ¿Hay días que podría dormir todo el tiempo?	No: 0 / Puntualmente: 1 / A veces: 2 / Siempre: 3
55. ¿Dormita durante el día?	No: 0 / Puntualmente: 1 / A veces: 2 / Siempre: 3
56. ¿Se siente con pocas ganas de hacer cosas?	No: 0 / Puntualmente: 1 / A veces: 2 / Siempre: 3
